# New Drugs and Preparations

**Published:** 1895-02-09

**Authors:** 


### NEW DRUGS AND PREPARATIONS.

[We shall be glad to receive, at our Office, 428, Strand, London, W.C., from the manufacturers, specimens of all new preparations and drugs which may be brought out from time to time.]

### TANNIGEN: AN INTESTINAL ASTRINGENT.

Tannigen is a derivative of tannic acid obtained by introducing into it two molecules of acetyl. It is a a yellowish-grey, odourless, tasteless powder, melting at 190 deg. C., and softening under the action of hot water into a mass of honey-like consistence. It is insoluble in cold water, sparingly in hot, freely soluble in alcohol, and in sodium phosphate, carbonate, or biborate. If left in contact with alkaline solutions for some days it is decomposed into acetic and gallic acids. It has been proposed by Meyer as a substitute for tannin in the treatment of diarrhœa, the advantage claimed for it being that it is not dissolved in the acid fluids of the stomach and passes through the latter without exercising any disturbing or astringent effect on its mucous membrane. Experiments on rabbits showed that considerable quantities had no effect on the stomach, while there was a very distinct astringent action exercised on the intestine, the fæces becoming hard and scanty. Müller has found it valuable in chronic diarrhœa, especially when of tubercular origin, in doses of three to eight grains, although it may be given in much larger amounts, even up to 60 grains in the twenty-four hours, without causing any hurtful effects. In cases of acute and of infantile diarrhœa it proved of little value, but in these an astringent treatment is seldom the most appropriate. Apparently it does not injure appetite nor gastric digestion. As a local astringent in pharyngitis a 3 to 5 per cent. solution in sodium phosphate acted well.

Deutsche Med. Wochenschr, 1894, 626.

